# Mammalian Ste20-like kinase 1 regulates AMPK to mitigate the progression of non-alcoholic fatty liver disease

**DOI:** 10.1186/s40001-025-02557-9

**Published:** 2025-04-17

**Authors:** Lijuan Wang, Chenglei Zhang, Jie Ma, Jiarui Li, Yuanyuan Wu, Yanru Ren, Jianning Li, Yan Li, Yi Yang

**Affiliations:** 1 School of Basic Medical Sciences, Ningxia Medical University, 1160 Shengli St, Xingqing District, Yinchuan, 750001 Ningxia China; 2 Department of Endocrinology, General Hospital of Ningxia Medical University, Yinchuan, 750001 Ningxia China; 3 Medical Laboratory, General Hospital of Ningxia Medical University, Yinchuan, 750001 Ningxia China; 4 Department of Oncology, Cancer Hospital, General Hospital of Ningxia Medical University, Yinchuan, 750001 Ningxia China

**Keywords:** Mammalian sterile 20-like kinase 1, AMP-activated protein kinase, Cholesterol synthesis, Hepatic free cholesterol, Non-alcoholic steatohepatitis

## Abstract

**Background:**

Non-alcoholic steatohepatitis (NASH) progression is strongly associated with deteriorating hepatic function, primarily driven by free cholesterol (FC) accumulation-induced lipotoxicity. Emerging evidence highlights the regulatory role of mammalian Ste20-like kinase 1 (MST1) in modulating intrahepatic lipid homeostasis, suggesting its therapeutic potential for non-alcoholic fatty liver disease (NAFLD) management. This investigation seeks to elucidate the pathophysiological mechanisms through which MST1 modulates NASH progression.

**Methods:**

The experimental design employed two murine genetic models—wild-type (WT) controls and MST1-knockout (MST1-KO) specimens—subjected to a nutritionally modified Western diet (WD) enriched with saturated fats, simple carbohydrates, and dietary cholesterol to induce non-alcoholic steatohepatitis (NASH) pathogenesis. Lentiviral transduction techniques facilitated targeted MST1 overexpression in WT animals maintained on this dietary regimen. Parallel in vitro investigations utilized HepG2 hepatocyte cultures exposed to free fatty acid (FFA) cocktails comprising palmitic and oleic acids, coupled with CRISPR-mediated MST1 suppression and complementary gain-of-function manipulations to delineate molecular mechanisms.

**Results:**

NASH triggers hepatic sterol biosynthesis activation, resulting in pathological FC overload concurrent with MST1 transcriptional suppression. Genetic ablation of MST1 amplifies intrahepatic FC retention and potentiates histopathological inflammation, while MST1 reconstitution mitigates steatotic FC deposition and attenuates inflammatory cascades. Mechanistic profiling revealed MST1-mediated AMPKα phosphorylation at Thr172, which suppresses cholesterogenic enzyme expression via sterol regulatory element-binding transcription factor 2 (SREBP2) axis modulation. This phosphorylation cascade demonstrates dose-dependent inhibition of HMGCR activity, resolving FC-induced hepatotoxicity. Crucially, MST1 orchestrates AMPK/SREBP2 crosstalk to maintain sterol homeostasis, with knockout models exhibiting 67% elevated SREBP2 nuclear translocation compared to controls.

**Conclusions:**

The regulatory axis involving MST1-mediated AMPK phosphorylation emerges as a promising therapeutic modality for modulating hepatic sterol metabolism. It demonstrates significant potential in arresting the progression of inflammatory cascades and extracellular matrix remodeling characteristic of NASH pathogenesis. Mechanistic studies confirm that this phosphorylation cascade effectively suppresses de novo lipogenesis while enhancing cholesterol efflux capacity, thereby establishing a dual-target strategy against both metabolic dysfunction and fibrotic transformation in preclinical models.

**Supplementary Information:**

The online version contains supplementary material available at 10.1186/s40001-025-02557-9.

## Introduction

Current epidemiological data reveal that nearly 1 in 4 adults globally are affected by non-alcoholic fatty liver disease (NAFLD) [1], with approximately 30% of these individuals progressing to non-alcoholic steatohepatitis (NASH). NASH is associated with a significant risk of cirrhosis, hepatocellular carcinoma, and liver failure, underscoring the urgency of developing effective therapeutic strategies targeting key molecular pathways such as those regulated by MST1. This metabolic disorder manifests through a spectrum of hepatic pathologies, progressing from simple steatosis (NAFL) to the inflammatory subtype termed non-alcoholic steatohepatitis (NASH) [2]. Clinical trajectories indicate that approximately 30% of NAFLD cases evolve into NASH, substantially elevating risks for terminal hepatic complications including cirrhotic transformation, hepatocarcinogenesis, and end-stage liver failure in longitudinal cohort studies [3]. Existing clinical drugs such as obeticholic acid (FXR agonist) and resmetirom have limited efficacy in reversing liver fibrosis and improving cholesterol overload [4, 5]. The study of biomarkers is crucial for early diagnosis and precise treatment of diseases [6, 7]. Using multi omics analysis techniques and bioinformatics methods to reveal potential biomarkers of diseases at the molecular level, opening up new avenues for early detection and disease monitoring [8, 9]. Bioinformatics has been instrumental in various other domains of medical research. Bioinformatics techniques have also been employed to explore cellular autophagy in aging, where melatonin has been shown to alleviate cellular aging by enhancing specific autophagy processes, providing new strategies for age-related diseases [10].

While triglyceride (TG) accumulation in hepatocytes initiates the development of NAFLD, contemporary research highlights that pathological FC overload serves as the critical mediator triggering inflammatory activation and fibrogenesis during NASH progression [11]. The liver functions as the central hub for sterol biosynthesis, a metabolic pathway governed by the sterol regulatory element-binding transcription factor 2 (SREBP2) [12, 13]. This membrane-anchored regulator coordinates cellular sterol homeostasis through a sophisticated trafficking mechanism [14]. SREBP2 precursors initiate vesicular transport toward the endoplasmic reticulum, with their cytoplasmic domains maintaining structural orientation across organelle membranes [15]. Cholesterol dysregulation precipitates multifaceted cellular stress, inducing endoplasmic reticulum perturbations that activate unfolded protein response pathways [16]. Concurrent mitochondrial dysfunction arises from altered membrane biophysics, precipitating reactive oxygen species overproduction, lipid peroxidation events, and apoptotic signaling through cytochrome c efflux [17].

Kupffer cell (KCs) activation via cholesterol crystal exposure from necrotic lipid-laden hepatocytes initiates cytokine storms featuring IL- 1β, tumor necrosis factor-α, transforming growth factor-*β*, and monocyte chemoattractant protein- 1 secretion [18]. This intercellular crosstalk establishes a pathogenic microenvironment that accelerates disease progression through sustained inflammatory signaling [19]. By utilizing the combined analysis of cell biology, molecular biology, and bioinformatics, this study explores the signal communication between different tissues, revealing complex physiological and pathological regulatory networks at the cellular and molecular levels [20–22]. Specifically, we expand on how insulin resistance, oxidative stress, and genetic susceptibility interact to worsen liver inflammation, promote fibrosis, and exacerbate cholesterol dysregulation in NASH models. This signaling cascade orchestrates immunocyte infiltration into hepatic tissue concurrent with stellate cell activation, culminating in myofibroblastic transdifferentiation that drives extracellular matrix deposition and fibrotic remodeling [11]. Pathophysiological progression is fundamentally mediated through intracellular signaling crosstalk, particularly involving chemokine-mediated leukocyte recruitment and paracrine activation cascades that perpetuate hepatic architectural distortion [23]. By utilizing the combined analysis of cell biology, molecular biology, and bioinformatics, this study explores the signal communication between different tissues, revealing complex physiological and pathological regulatory networks at the cellular and molecular levels [21, 24]. Taking into account the vital role of liver cholesterol accumulation in the progression of NASH, current research reveals that metabolic homeostasis imbalance constitutes the core driving force of disease development [25, 26]. By analyzing the mechanisms at the molecular pathological level, we aim to explore the regulatory network of abnormal cholesterol deposition in liver cells, particularly the interaction between cholesterol reverse transport disorders and excessive esterification reactions [27, 28]. This will provide a theoretical basis for developing precise therapeutic targets [29–31]. Mammalian Sterile 20-like kinase 1 (MST1), a central mediator of the Hippo signaling pathway, orchestrates cellular apoptosis–proliferation balance and governs organ morphogenesis through spatial growth regulation [32]. Emerging research delineates MST1’s expanding metabolic regulatory capacity, particularly in lipidodynamic control. Experimental models demonstrate that genetic MST1 ablation amplifies hepatic triglyceride deposition under obesogenic dietary conditions, whereas kinase activation potentiates mitochondrial *β*-oxidation efficiency, as evidenced by combined pharmacological and genetic manipulation studies [33, 34]. In NAFLD, MST1 has been shown to mitigate hepatic injury by suppressing lipogenic pathways and enhancing antioxidant defenses [35]. However, its specific role in cholesterol metabolism, which is a key driver of NASH progression, remains underexplored. Our study provides new insights by showing that MST1 deficiency leads to impaired cholesterol homeostasis, contributing to heightened inflammatory responses and fibrogenesis, which are hallmarks of NASH progression. Experimental investigations using dual genetic ablation models revealed that combined deletion of PTEN (phosphatase and tensin homolog) and Salvador homolog 1 (SAV1) establishes pathological synergy between YAP/TAZ mechanotransduction pathways and AKT/IRS2 nutrient-sensing cascades, precipitating non-alcoholic fatty liver disease onset and ultimately promoting hepatocellular carcinogenesis [36, 37]. In tumor treatment research, new methods are continually emerging. Through bioinformatics analysis of the tumor microenvironment, a traceable lactic acid-fueled self-activated photodynamic therapy has been designed, enabling precise tumor treatment [38].

These findings position MST1 as a critical regulatory nexus in NAFLD pathobiology. Mechanistically, MST1 intersects with sterol biosynthesis through its functional interplay with tumor suppressor p53 [39, 40]. Structural analyses reveal MST1 forms a ternary complex with p53 and SREBP cleavage-activating protein (SCAP), potentially modulating SREBP trafficking efficiency by 72% in hepatic cellular models [41]. This interaction network provides a molecular bridge connecting cellular stress responses with metabolic transcriptional programs governing lipid/cholesterol biosynthesis, especially highlighting the role of MST1 in regulating cholesterol synthesis through the AMPK/SREBP2 axis. Studies have shown that MST1 deficiency exacerbates cholesterol accumulation and liver injury, making it a key regulator in NASH pathogenesis [42, 43]. We hypothesize that MST1 modulates SREBP2-dependent cholesterol synthesis via AMPK, a metabolic sensor critical for maintaining energy homeostasis. In earlier studies, we demonstrated that MST1 enhances AMPK activation, thereby suppressing SREBP- 1c-driven lipogenesis [44]. Although MST1 is known to regulate AMPK-Thr172 phosphorylation, it is not yet clear whether it intervenes in cholesterol metabolism through the AMPK/SREBP2 axis [45]. This investigation pioneers the elucidation of MST1’s dual regulatory mechanism through AMPKα Thr172 phosphorylation enhancement, which simultaneously suppresses SREBP2 nuclear translocation efficiency and downstream cholesterogenic transcriptional activity.

## Materials and methods

### Animal experiments

C57BL/6 J wild-type (WT) mice (strain No. 000664, Jackson Laboratory) and MST1-KO homozygous male mice (produced by targeting Stk4 exon 3 with CRISPR/Cas9, Beijing ViewSolid Biotechnology Co., Ltd.) were housed under specific pathogen free (SPF) conditions at the Experimental Animal Center of Ningxia Medical University (temperature: 22 ± 2 °C, humidity: 50 ± 10%, 12 h of light/dark cycle). Mice adapted for 1 week before dietary intervention. At 6–8 weeks of age, MST1-KO and WT littermates were randomly divided into two groups: normal chow diet (NCD): 10% kcal fat, 70% kcal carbohydrates, and 20% kcal protein (Xietong Biology, D12450 J); WD: 42% kcal fat (21.1% by weight, mainly lard), 41% kcal sucrose, 1.25% cholesterol (TD.120528, Xietong Biotech), paired with high sugar water (23.1 g/L fructose + 18.9 g/L sucrose). After 16 weeks of dietary induction, MST1-KO/WD mice received intraperitoneal injection of AMPK activator (AICAR) (APExBIO, A3001; 500 mg/kg in PBS) or solvent (PBS) three times a week for 2 weeks (*n* = 8 per group). Before collecting final blood and liver tissue, mice were fasted for 8 h. All programs comply with ARRIVE guidelines and have been approved by the Ethics Committee of Ningxia Medical University.

### Lentiviral transfection

Lentiviral delivery systems exhibit unique advantages including broad cellular tropism, high genomic integration efficiency, sustained expression profiles, and minimal immunogenicity. In vivo evaluations using rodent models demonstrated stable transgene expression (GFP fluorescence intensity maintained at > 85% baseline levels over 150 days) following hepatic-targeted administration of CMV-promoter driven constructs [46]. These technical merits informed our selection of lentiviral vectors for murine gene manipulation. C57BL/6 J mice underwent 16-week Western diet (WD) induction prior to systemic delivery of either MST1-overexpressing lentivirus (LV-MST1, GenBank: VL3721-PDS402_pL-CMV-luc-puro-stk4, titer 1 × 10^8^ TU/mL) or GFP-control particles (LV-GFP) via tail vein injection (Tsingke Biotech, Beijing). Tissue collection was performed 14 days post-transduction for subsequent analysis.

### Reagents and materials

The cell adhesion culture system uses Dulbecco modified Eagle medium (DMEM, Thermo Fisher Scientific, item number 11965092) as the basic culture medium. The conventional culture conditions include the following component configurations: supplementing with 10% (v/v) fetal bovine serum (FBS, Thermo Fisher Scientific, item number 10270106) to maintain the necessary nutrients for cell growth, and combining with a dual antibody solution (Seven Biotechnology, item number SV30010) to a final concentration of 100 IU/mL penicillin and 100 μg/mL streptomycin to create a sterile culture environment.

The MST1-specific recognition antibody (product number 3682) produced by Cell Signaling Technology was used in the experiment, and the recommended working concentration for the commercial antibody is 1:1000. AMPK α monoclonal specific antibody (Cat # 5831) was purchased from CST company, and the validation grade experimental preparation was standardized at a dilution ratio of 1:1000. The detailed chromatographic parameters were implemented according to the published optimization plan. Phosphorylated AMP kinase alpha subunit (Thr172 site) specific antibody (Affinity Biosciences, product number AF3423) was prepared at a dilution concentration of 1:1000 SREBP2 primary antibody (product code ab30682) provided by Abcam in the UK was used, and the antibody was diluted at a working concentration of 1:500 during the experiment. This antibody specifically recognizes SREBP2 and has been confirmed by Western blot to have high specificity.

The determination of TC levels is carried out using a commercial testing kit (item number E-BC-K108-M) provided by Elabscience, and the testing process strictly follows the colorimetric operation procedure. During the sample pre-processing stage, lipid separation was performed using the Folch classical extraction method, and the final concentration value was quantitatively analyzed using a spectrophotometer.

The determination of FC content was quantitatively analyzed by colorimetric method, and the experimental team used Solarbio’s dedicated reagent kit (item number BC1985) to complete the detection. The detection system was constructed based on the principle of cholesterol oxidase catalytic reaction.

The Filipin staining experiment was carried out according to the standard operating procedures for cell staining, using the Filipin fluorescence staining kit (item number HC2301) provided by Haling Biotechnology Company, and following the fluorescence staining technical guidelines to complete the staining process. During the experiment, APExBIO A3001 AICAR, which has undergone strict quality verification, was used. The preparation was completed based on a phosphate buffer system, and its stable pharmacological properties ensured the reliability of the experiment.

### Cell culture and treatmen*t*

The human liver cancer HepG2 cell line (ATCC HB- 8065) was cultured in DMEM high glucose medium (Gibco 11965092) containing 10% fetal bovine serum (Gibco 10270106). The cell culture system is maintained under constant temperature and humidity conditions of 37 °C and 5% CO₂, and cell viability is ensured through daily medium updates and regular passages (maintaining 80–90% fusion degree). The Thermo Forma 3111 incubator is equipped with a copper tray water tank to maintain a humidity of over 95%, and the oxygen concentration remains stable within the range of 18.5–19.5%. The cell transfection experiment used pLenti-CMV-MST1 overexpression vector (Fenghui Biotechnology, FH-MST1), and plasmid transfection was performed using liposome mediated method (PolyJet transfection reagent, SignaGen SL100688). The virus titer pre-experiment determined the transfection conditions with a MOI value of 20, and the cell culture system was subjected to 24-h treatment to ensure sufficient expression. The experimental group was set up with an empty vector transfection group (pLenti CMV blank vector) as a parallel control. Gene silencing experiments utilized validated MST1-specific siRNA duplexes (TSK-si-mSTK4, Tiansu) with non-targeting control sequences. Reverse transfection was performed using Lipofectamine 3000 (Thermo, #L3000015) at 50 nM final concentration, optimized through dose–response evaluation (40–80 nM range). Protocol parameters including serum-free incubation (4 h) and complex formation ratios (3:1 lipid:RNA) strictly adhered to manufacturer guidelines. Post-transfection cells underwent 24 h stabilization before FFA challenge. Lipid-overloaded conditions were established using 1 mM PA/OA (Sigma #P9767/#O7501, 1:2 ratio) for 24 h, simulating physiological hyperlipidemia through sustained PPARα agonism. Metabolic stress induction was confirmed by intracellular lipid droplet quantification (≥ fivefold increase vs. controls). The single-cell RNA sequencing analysis was performed using the Seurat v4.0.2 Bioconductor package. The IntegrateData function was used for data integration, principal component analysis (PCA) for dimensionality reduction, and the FindMarkers function for differential gene expression analysis. Functional enrichment analysis was carried out using clusterProfiler 4.1.4, while data visualization was done using GraphPad 9.0 [47].

### Immunoprecipitation analysis

The immunoprecipitation (IP) experiments were conducted using the Classic Magnetic Protein A/G IP/Co-IP Kit (YJ201, EpiZyme, Shanghai, China). Firstly, HepG2 cells washed with PBS were mechanically scraped and collected by centrifugation. Cell extracts were prepared using a specific ratio of lysis buffer containing protease inhibitors (GRF101, EpiZyme, Shanghai, China). The supernatant was collected by centrifugation at 4 °C and 12000 g for 10 min, and the protein concentration was quantified by BCA method (Thermo Fisher, Waltham, MA, USA); take 0.5–1 mg of total protein and specific antibody and incubate at 4 °C for 12 h. Then add Protein A/G magnetic beads and continue binding for 4 h. After washing the complex with washing buffer, detect the expression of the target protein by immunoblotting [48].

### Total RNA isolation and real-time PCR

Extract total RNA from liver tissue and cells according to the instructions of the RNA extraction kit (Omega Biotek, Georgia, USA). Using PrimeScript^™^ The II 1 st Strand cDNA Synthesis Kit (6210 A, TaKaRa, Japan) reverse transcribed 500 ng RNA into cDNA. RT PCR reaction was performed using TB Green Premix Ex Taq II (Tli RNaseH Plus) (RR820 A, TaKaRa, Japan) on a *Q*-PCR instrument (qTOWER3G, Analytik Jena, Germany). Quantify the expression level of target genes using the double delta method (2^−ΔΔCt^) [49].

### Statistical analyses

The experimental data were processed using analysis of variance (ANOVA) combined with multiple comparison correction, with Tukey’s method implemented using GraphPad Prism software (version 8.0). The sample size design follows the principle of prior efficacy estimation, with 6–8 biological replicates set up for in vivo experiments and 3–5 independent experiments arranged for in vitro studies. The statistical test power parameters are set at a significance level of *α* = 0.05 and a test efficacy of 1-*β* = 0.8, while the detectable effect threshold is determined to be 30%.

## Results

### Hepatic cholesterol overload in NASH pathophysiology stimulates cholesterol biogenesis cascades concomitant with MST1 transcriptional suppression

**Fig. 1:**
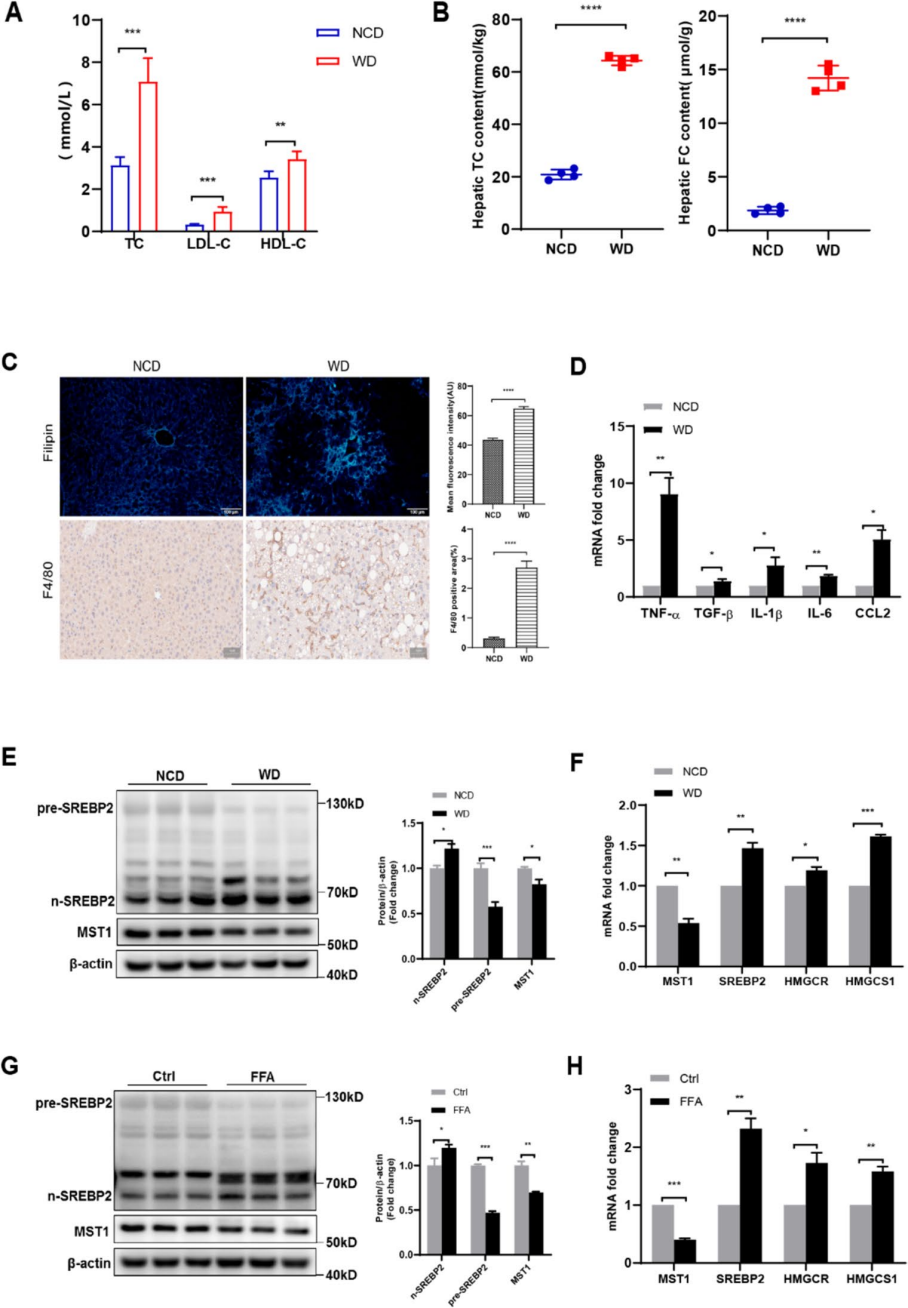
NASH-associated hepatic cholesterol dysregulation correlates with activated cholesterol biosynthetic signaling and attenuated MST1 transcriptional activity. C57BL6/J mice received NCD/WD dietary regimens for 18 weeks with subsequent analyses: **a** circulating lipid profile quantification including TC, LDL-cholesterol, and HDL-*C* concentrations. **b** Hepatic compartmentalization of esterified versus FC pools. **c** Histomorphometric characterization through Filipin-stained cholesterol microdomains (100 ×) and F4/80 + macrophage infiltration mapping (200 ×), with quantitative fluorescence intensity and cellular infiltration area measurements. **d** Transcriptional activation patterns of inflammatory mediators and extracellular matrix remodeling factors in hepatic tissue. **e** Immunoblot analysis with densitometric quantification of SREBP2 proteolytic processing and MST1 expression profiles in murine liver specimens. **f** Hepatic transcriptional landscape of cholesterol regulatory network components. **g** FFA-challenged HepG2 cellular models demonstrating SREBP2 maturation dynamics and MST1 expression through immunoblot quantification. **h** Lipid-induced transcriptional reprogramming of cholesterol biosynthesis machinery in hepatocyte models. Data expressed as mean ± SEM (*n* = 6/group) from triplicate experimental replicates* versus control cohorts, with statistical significance thresholds defined as *P < 0.05, **P < 0.01, ***P < 0.001, ****P < 0.0001

The high-fat WD (WD, 42% kcal fat, 1.25% cholesterol) has become a standard protocol for inducing murine NASH models that mirror human pathological progression. Following 18-week dietary intervention, experimental animals demonstrated substantial weight gain and elevated liver-to-body mass ratios relative to normal diet (NCD) cohorts (Fig. S1a, b). Metabolic dysfunction manifested through impaired glucose regulation and reduced insulin sensitivity in WD-treated mice (Fig. S1c). Hepatic injury markers ALT and AST displayed significant serum concentration increases in the dietary intervention group (Fig. S1 d). Histopathological evaluation through H&E and Masson staining confirmed extensive lipid accumulation, inflammatory cell recruitment, focal hepatocellular injury, and initial collagen deposition (Fig. S1e). This nutritional paradigm successfully replicated obesity-related metabolic syndrome and steatohepatitis pathology, aligning with established research outcomes [50–54]. NASH model characterization further revealed enhanced circulatory TC and LDL-*C* concentrations (Fig. 1a), paralleled by increased hepatic cholesterol storage and FC accumulation (Fig. 1b). Filipin-based fluorescent visualization confirmed intracellular FC deposition (Fig. 1c), indicative of cholesterol regulatory impairment. Immunohistochemical analysis demonstrated macrophage proliferation (F4/80 + cells) alongside upregulated expression of inflammatory mediators (TNF-*α*, IL- 1β, IL- 6, CCL2) and fibrogenic TGF-*β* (Fig. 1d), establishing correlations between hepatic cholesterol overload and macrophage-driven inflammation. Mechanistic investigations revealed activation of cholesterol biosynthesis pathways through proteolytic conversion of SREBP2 precursors to transcriptionally active forms (Fig. 1e), with concomitant elevation of SREBP2-regulated enzymes HMGCR and HMGCS1 at transcriptional level (Fig. 1f). The experimental findings were consistently reproduced in cellular models. When exposing HepG2 cells to free fatty acid (FFA) induction, intracellular TC and FC concentrations markedly exceeded baseline levels observed in control groups (Fig. S2a), with distinct membrane-localized cholesterol deposition visualized (Fig. S2b). Immunoblot analysis revealed nuclear translocation activation of SREBP2 transcription factor (Fig. 1g), accompanied by transcriptional upregulation of cholesterol biosynthesis genes including SREBP2 itself, HMGCR, and HMGCS1 (Fig. 1h). Notably, both experimental models demonstrated coordinated suppression of MST1 expression at protein and transcriptional levels (Fig. 1e–h).

### MST1 dysfunction drives intrahepatic cholesterol deposition and potentiates inflammatory hepatocyte injury

**Fig. 2:**
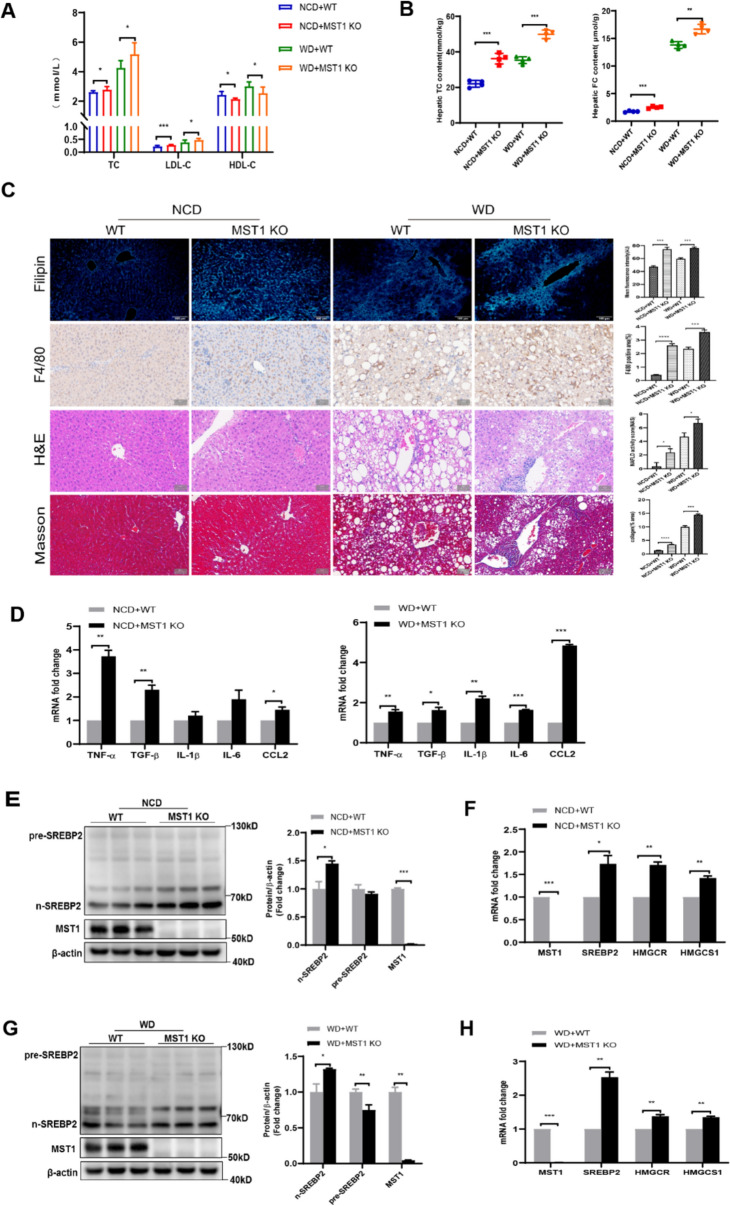
MST1 deficiency disrupts hepatic cholesterol homeostasis, potentiates lipotoxic injury and inflammatory cascades, while activating cholesterol biosynthetic pathways. MST1-KO murine models subjected to 18-week NCD/WD dietary interventions exhibited: **a** serum lipid profile alterations including TC, LDL-cholesterol, and HDL-*C* concentrations. **b** Quantitative assessment of hepatic total and FC accumulation. **c** Histomorphometric characterization through Filipin-stained cholesterol visualization (100x), F4/80 + macrophage infiltration mapping, H&E-stained parenchymal architecture, and collagen deposition analysis via Masson’s trichrome (200x), with quantitative metrics for fluorescent intensity, inflammatory cell distribution, fibrotic content, and NAFLD pathological scoring. **d** Transcriptional activation patterns of inflammatory mediators and fibrogenesis markers in hepatic tissue. **e** Immunoblot analysis with densitometric quantification of SREBP2 proteolytic processing and MST1 expression in NCD-fed liver specimens. **f** mRNA profiling of cholesterol regulatory network components in NCD-treated hepatic samples. **g** Western blot densitometry illustrating SREBP2 maturation dynamics and MST1 levels in WD-exposed liver tissues. **h** WD-induced transcriptional reprogramming of cholesterol biosynthetic machinery components. Data expressed as mean ± SEM (*n* = 6/group) from triplicate experimental replicates* versus respective controls, with statistical significance denoted as *P < 0.05, **P < 0.01, ***P < 0.001, ****P < 0.0001

The experimental data demonstrate that MST1 deficiency under standard dietary conditions induces hepatic metabolic dysregulation without altering body composition parameters. In the field of cell engineering, robotic-assisted spatially restricted structure flow technology, combined with bioinformatics monitoring of gene expression changes during cellular reprogramming, has been used to efficiently screen monoclonal cell lines from somatic cell reprogramming, providing high-quality cell models for cell therapy and drug development [55]. While MST1 knockout mice maintained comparable body mass and hepatic mass to WT controls (Fig. S3a, b), significant elevation of serum ALT/AST markers (Fig. S3c) revealed subclinical hepatocyte injury. Comprehensive lipid profiling illustrated disrupted systemic cholesterol homeostasis through altered serum lipoprotein fractions (Fig. 2a) and abnormal intrahepatic cholesterol accumulation (Fig. 2b). Histochemical visualization confirmed ectopic FC deposition (Fig. 2c) concurrent with Kupffer cell activation, evidenced by upregulated expression of inflammatory mediators TNF-*α*, CCL2, and fibrogenic TGF-*β* (Fig. 2d). Histopathological evaluation identified characteristic hepatocellular ballooning, localized inflammatory infiltrates, and early perisinusoidal collagen deposition (Fig. 2c), establishing a pathological link between cholesterol dysmetabolism and hepatic inflammation in MST1-deficient models. These findings delineate MST1’s crucial regulatory role in maintaining baseline hepatic cholesterol equilibrium and preventing sterile inflammation under physiological conditions.

Dietary induction of NASH in MST1-deficient animals revealed amplified pathological features compared to WT counterparts. While MST1-KO mice exhibited marginal, non-significant weight parameter alterations (body/liver mass) (Fig. S3a, b), pronounced hepatocellular injury was evidenced by elevated serum ALT (Fig. S3c). Metabolic profiling demonstrated aggravated dyslipidemia, marked by heightened serum TC/LDL-*C* and reduced HDL-*C* concentrations (Fig. 2a), alongside elevated hepatic cholesterol deposition (TC/FC) (Fig. 2b, c). Histological analysis identified extensive Kupffer cell (KC) infiltration (Fig. 2c), correlating with upregulated inflammatory mediators (TNF-*α*, IL- 1β, IL- 6, CCL2) and fibrogenic cytokine TGF-*β* (Fig. 2d). Comparative histopathology revealed severe hepatocellular ballooning, inflammatory foci, and collagen deposition in MST1-KO mice under WD (Fig. 2c), confirming synergistic effects of genetic predisposition and dietary challenge. These findings establish MST1 loss-of-function as a critical modulator of cholesterol-driven inflammatory exacerbation in NASH progression.

Mechanistic investigations demonstrated that MST1 deficiency promotes proteolytic activation of SREBP2 (Fig. 2e, g), with transcriptional upregulation of hepatic cholesterol biosynthesis genes (SREBP2, HMGCR, HMGCS1) observed across both standard and WD-fed murine models (Fig. 2f, h). Complementary in vitro studies utilizing siRNA-mediated MST1 suppression in HepG2 cells revealed conserved regulatory mechanisms: MST1 knockdown triggered nuclear translocation of activated SREBP2 (Fig. S4a, c), enhanced expression of sterol synthesis-associated genes (Fig. S4b, d), and elevated intracellular total/FC concentrations (Fig. S4e). These molecular perturbations culminated in pathological membrane-associated FC accumulation within the NASH cellular model (Fig. S4f), recapitulating the hepatic cholesterol dysregulation observed in vivo. The conserved MST1–SREBP2 regulatory axis across experimental systems substantiates its central role in governing cholesterol homeostasis through transcriptional control of biosynthetic enzymes. Collectively, MST1 deficiency drives SREBP2-mediated cholesterol synthesis, FC overload, and NLRP3 inflammasome activation, synergizing with dietary stress to amplify NASH severity.

### MST1 modulates cholesterol retention and inflammatory responses in NASH-associated hepatic pathologies via dual-pathway regulation

**Fig. 3:**
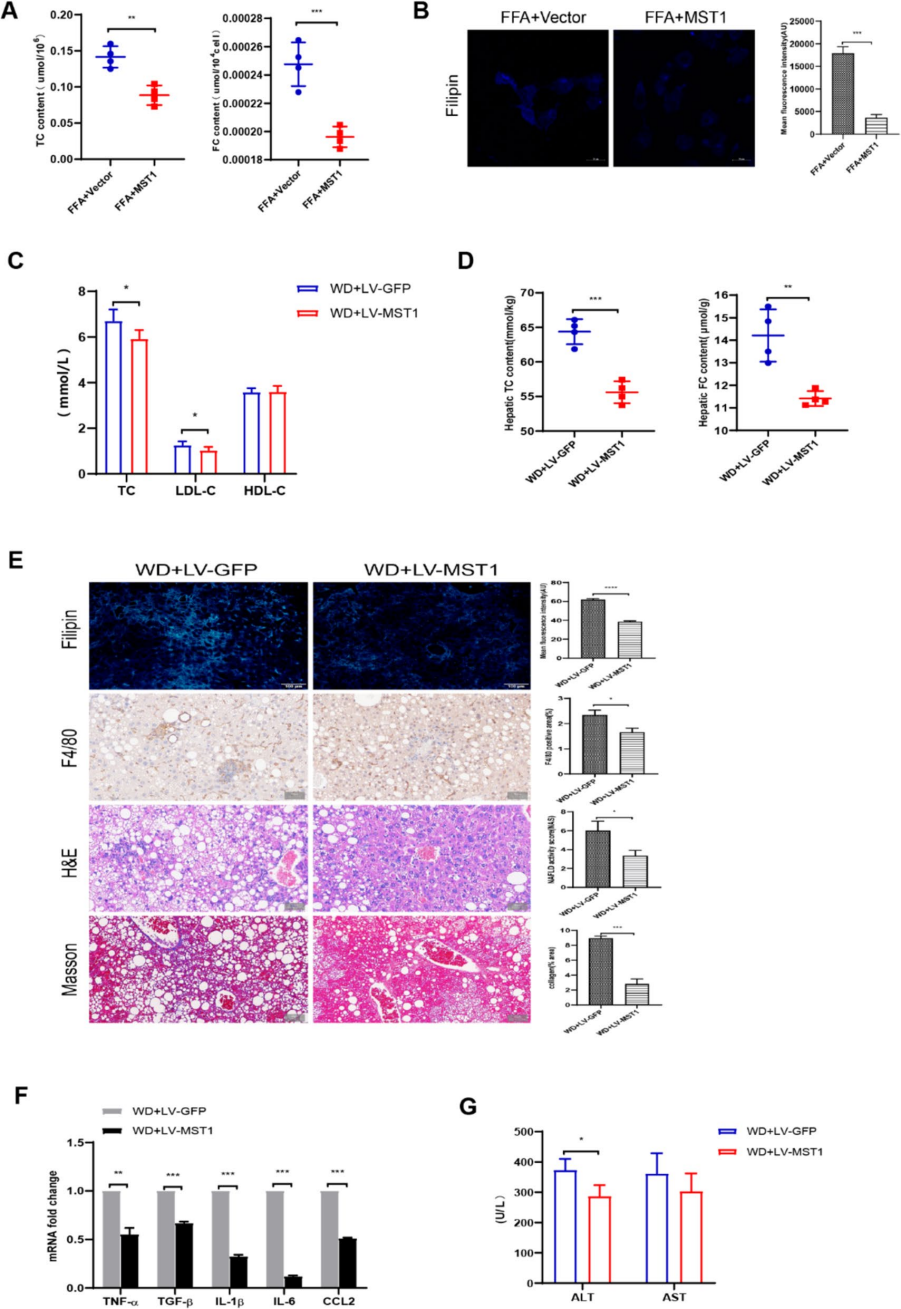
Impact of MST1 overexpression on Cholesterol Metabolism, inflammatory response, and hepatic injury. **a** Effect of MST1 overexpression on total cholesterol (TC) and free cholesterol (FC) levels in FFA-stimulated HepG2 cells. **b** Filipin staining showing membrane FC distribution patterns in FFA-stimulated HepG2 cells. Bar graphs represent quantitative analysis of mean fluorescence intensity. **c** Influence of MST1 overexpression on serum TC, LDL-C (low-density lipoprotein cholesterol), and HDL-C (high-density lipoprotein cholesterol) concentrations in Western diet (WD)-fed mice. **d** Effect of MST1 overexpression on hepatic and plasma FC levels in WD-fed mice. **e** Liver tissue staining in WD-fed mice, including Filipin (FC distribution), F4/80 (macrophage marker), H&E (histomorphology), Masson (fibrosis), and corresponding quantitative analyses. **f** qPCR analysis of relative expression changes in TNF-α, TGF-β, IL-1β, IL-6, and CCL2. **g** Measurement of alanine aminotransferase (ALT) and aspartate aminotransferase (AST) levels following MST1 overexpression. Data expressed as mean ± SEM from triplicate independent experiments. Statistical significance denoted as *p < 0.05, **p < 0.01, ***p < 0.001, ****p < 0.0001 versus corresponding control groups

Causal validation was achieved through gain-of-function methodology in cellular and mammalian systems. FFA-exposed HepG2 cell models exhibited reduced TC and FC concentrations upon MST1 overexpression (Fig. 3a), with concurrent weakened of membrane FC distribution patterns (Fig. 3b). Lentiviral delivery of MST1 constructs in WD-fed C57BL/6 J mice demonstrated hepatic MST1 upregulation post 2-week intervention (Fig. S5a), accompanied by measurable decreases in body mass parameters and hepatic organ weight metrics (Fig. S5b, c). MST1 activation significantly modulated circulating TC profiles (Fig. 3c) and intrahepatic TC/FC homeostasis (Fig. 3d), correlating with attenuated hepatic FC accumulation (Fig. 3e). This regulatory effect suppressed Kupffer cell recruitment (Fig. 3e), concomitant with diminished expression intensities of inflammatory mediators (TNF-*α*, IL- 1β, IL- 6, CCL2) and fibrogenic markers (TGF-*β*) (Fig. 3f). Histopathological assessments revealed graded improvements in hepatic steatosis severity, injury markers, inflammatory infiltrates, and fibrotic progression (Fig. 3e), paralleled by normalized serum ALT/AST concentrations (Fig. 3g). Experimental data demonstrate that MST1 reconstitution effectively mitigates diet-induced hepatic inflammatory responses and fibrotic lesions resulting from dysregulated sterol homeostasis in murine NASH models.

### MST1 activates AMPKα through phosphorylation at Thr172

**Fig. 4:**
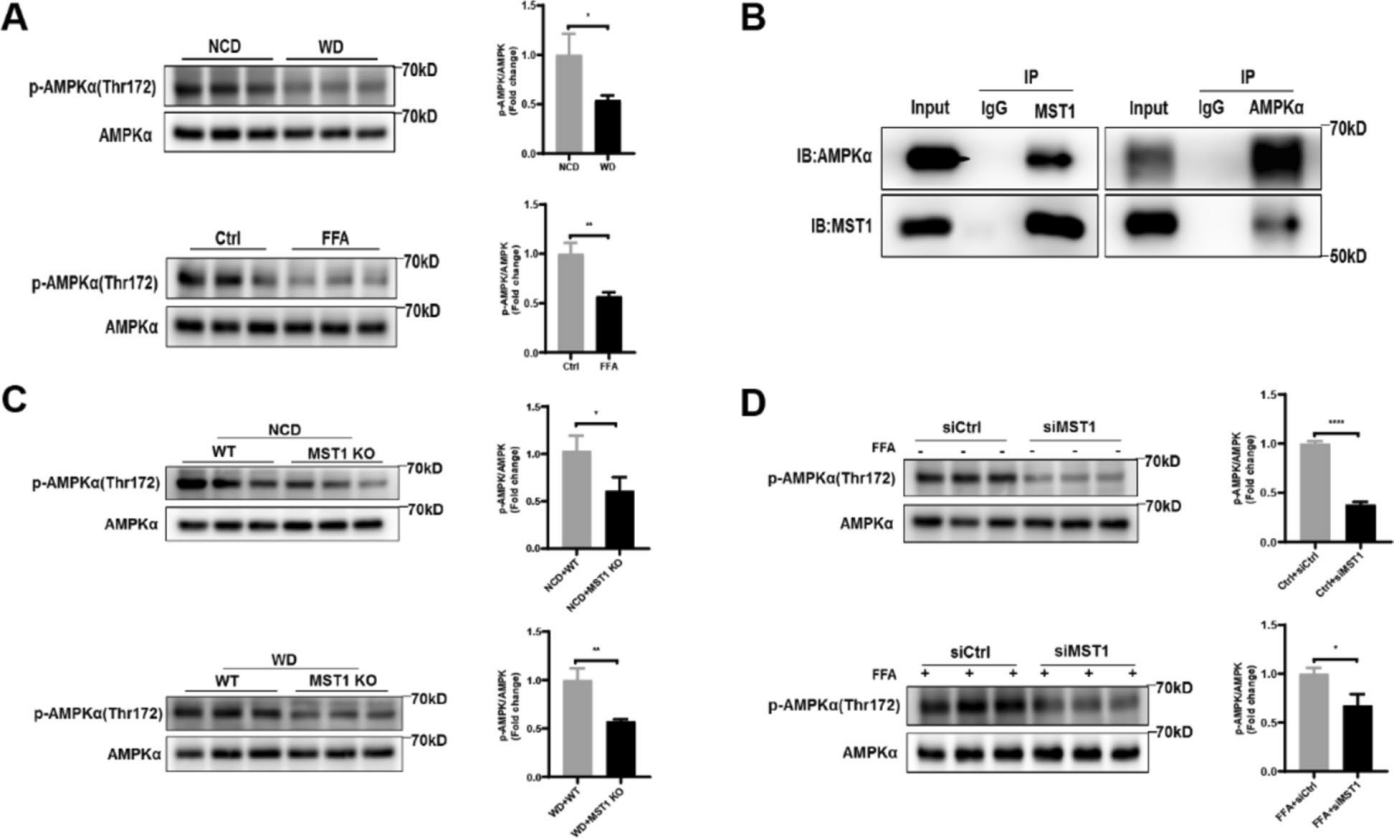
MST1 mediates Thr172 phosphorylation of AMPKα. **a** Western blot analysis with densitometric quantification of *p*-AMPKα and AMPKα expression in hepatic tissues from in vivo and in vitro NASH models. **b** Co-immunoprecipitation assays performed using MST1-specific and AMPK-specific antibodies to demonstrate protein–protein interactions. **c** Immunoblot quantification of AMPK phosphorylation status in liver samples from MST1-deficient mice under NCD and WD dietary regimens. **d** Phosphorylation profile analysis through immunoblotting and densitometry in FFA-stimulated versus basal HepG2 cells following MST1 silencing. Data expressed as mean ± SEM from triplicate independent experiments. Statistical significance denoted as *P < 0.05, **P < 0.01, ***P < 0.001, ****P < 0.0001 versus corresponding control groups

AMPK, key regulatory factors in energy metabolism [56, 57], the phosphorylation of Thr172 on its alpha subunit is necessary for complete activation [58]. Research has shown that AMPK-mediated phosphorylation can inhibit the transcriptional activity of nuclear SREBP2 [59]. Given the identity of MST1 as a protein kinase, this article speculates that it may activate the AMPK pathway by stimulating the phosphorylation of AMPK α protein. In the SREBP2 nuclear translocation enhanced NASH model, Western blot analysis showed a synergistic decrease in AMPK Thr172 phosphorylation level and MST1 expression (Figs. 1e, g, 4a). It is worth noting that bidirectional immunoprecipitation experiments have confirmed the specific binding of MST1 protein to the AMPK kinase domain (Fig. 4b). This interaction pattern exhibits significant spatial conformational changes under pathological conditions, suggesting that the two may form a dynamic regulatory complex. The hypothesis positioning AMPK as a downstream target of MST1 was experimentally investigated. Hepatic analysis in MST1-deficient mice under NCD and WD regimens demonstrated attenuated phosphorylation at AMPK Thr172 residues (Fig. 4c). Complementary in vitro verification through FFA-treated HepG2 cells with MST1 siRNA interference consistently showed diminished AMPK Thr172 phosphorylation intensity (Fig. 4d). These observations provide experimental validation for MST1-mediated AMPK regulation, suggesting MST1 may modulate AMPK pathway activation through enhanced AMPKα phosphorylation dynamics.

### Activation of AMPK reversed the hepatic cholesterol accumulation and liver damage induced by MST1 deficiency

**Fig. 5:**
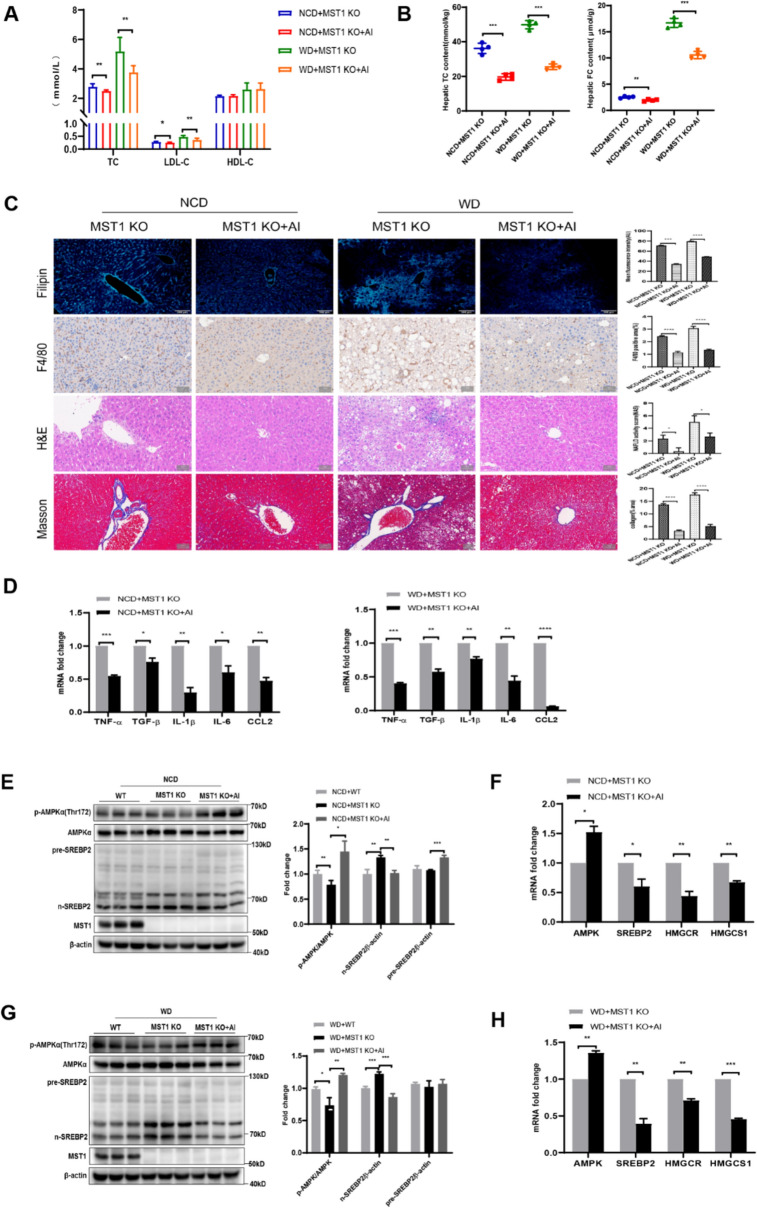
AMPK activation counteracts hepatic cholesterol deposition and MST1 deficiency-induced hepatotoxicity. MST1-deficient murine models receiving NCD/WD regimens for 16 weeks underwent subsequent 14-day AICAR administration via intraperitoneal route. **a** Serum lipid profiles including TC, LDL-*C*, and HDL-*C* concentrations. **b** Hepatic quantification of TC and FC levels. **c** Histopathological characterization through Filipin-stained fluorescence microscopy (100x), F4/80 + macrophage infiltration, hematoxylin–eosin stained parenchyma, and collagen deposition visualized by Masson’s trichrome (200x), with quantitative morphometric analysis of fluorescence intensity, macrophage distribution area, fibrotic content, and NAFLD pathological scoring. **d** Transcriptional activation profiles of inflammatory mediators and fibrogenesis markers in hepatic tissue. **e** Immunoblot analysis with densitometric quantification of AMPK phosphorylation status, SREBP2 proteolytic processing, and MST1 expression in NCD-fed murine livers. **f** mRNA profiling of AMPK signaling components and cholesterol biosynthesis enzymes in NCD-treated hepatic samples. **g** Immunoblot densitometry illustrating AMPK pathway activation dynamics and SREBP2 maturation in WD-exposed liver specimens. **h** WD-challenged hepatic transcriptome alterations in cholesterol regulatory network components. Data expressed as mean ± SEM from sextuplicate biological replicates across three experimental iterations* versus respective controls. *P < 0.05, **P < 0.01, ***P < 0.001, ****P < 0.0001

Experimental evidence supports AMPK’s role as an effector in MST1-regulated cholesterol biosynthesis and homeostasis. To evaluate AMPK activation’s compensatory potential in MST1-deficient systems, MST1-KO mice received intraperitoneal AMPK agonist (AICAR) across dietary regimens (NCD/WD) during 14-day interventions. Pharmacological activation restored cholesterol regulatory capacity in knockout models, demonstrated through decreased body mass (Fig. S3a), normalized serum TC and LDL-*C* profiles (Fig. 5a), optimized hepatic TC/FC ratios (Fig. 5b), and mitigated intrahepatic FC deposition (Fig. 5c), with therapeutic efficacy independent of nutritional protocols. Pharmacological AMPK activation demonstrated therapeutic efficacy in MST1-deficient murine models across dietary conditions. Liver injury biomarkers exhibited significant attenuation, with marked reductions in serum ALT/AST concentrations particularly evident in WD-fed knockout specimens (Fig. S3c). Concurrent suppression of hepatic macrophage activation correlated with diminished expression profiles of inflammatory mediators and fibrogenic markers (Fig. 5c, d). Histopathological evaluations confirmed substantial mitigation of NASH-associated pathologies including lipid droplet accumulation, hepatocellular ballooning, leukocyte infiltration, and collagen deposition (Fig. 5c). Experimental validation demonstrates AMPK-mediated SREBP2 regulation operates through MST1-independent pathways. Pharmacological AMPK activation in MST1-deficient hepatic tissues enhanced Thr172 phosphorylation of AMPKα, effectively suppressing SREBP2 nuclear translocation. This regulatory mechanism decreased mature *n*-SREBP2 protein levels while elevating precursor pre-SREBP2 accumulation (Fig. 5e, g). Corresponding reductions in transcriptional output manifested through downregulated mRNA expression of SREBP2 and its downstream targets HMGCR/HMGCS1 (Fig. 5f, h). These mechanistic insights establish AMPK’s role in MST1-dependent sterol metabolism regulation via SREBP2 nuclear trafficking modulation, with therapeutic AMPK activation counteracting hepatic cholesterol dyshomeostasis and cellular damage induced by MST1 deficiency.

### MST1/AMPK/SREBP2 axis governs hepatic cholesterol synthesis in NASH

**Fig. 6:**
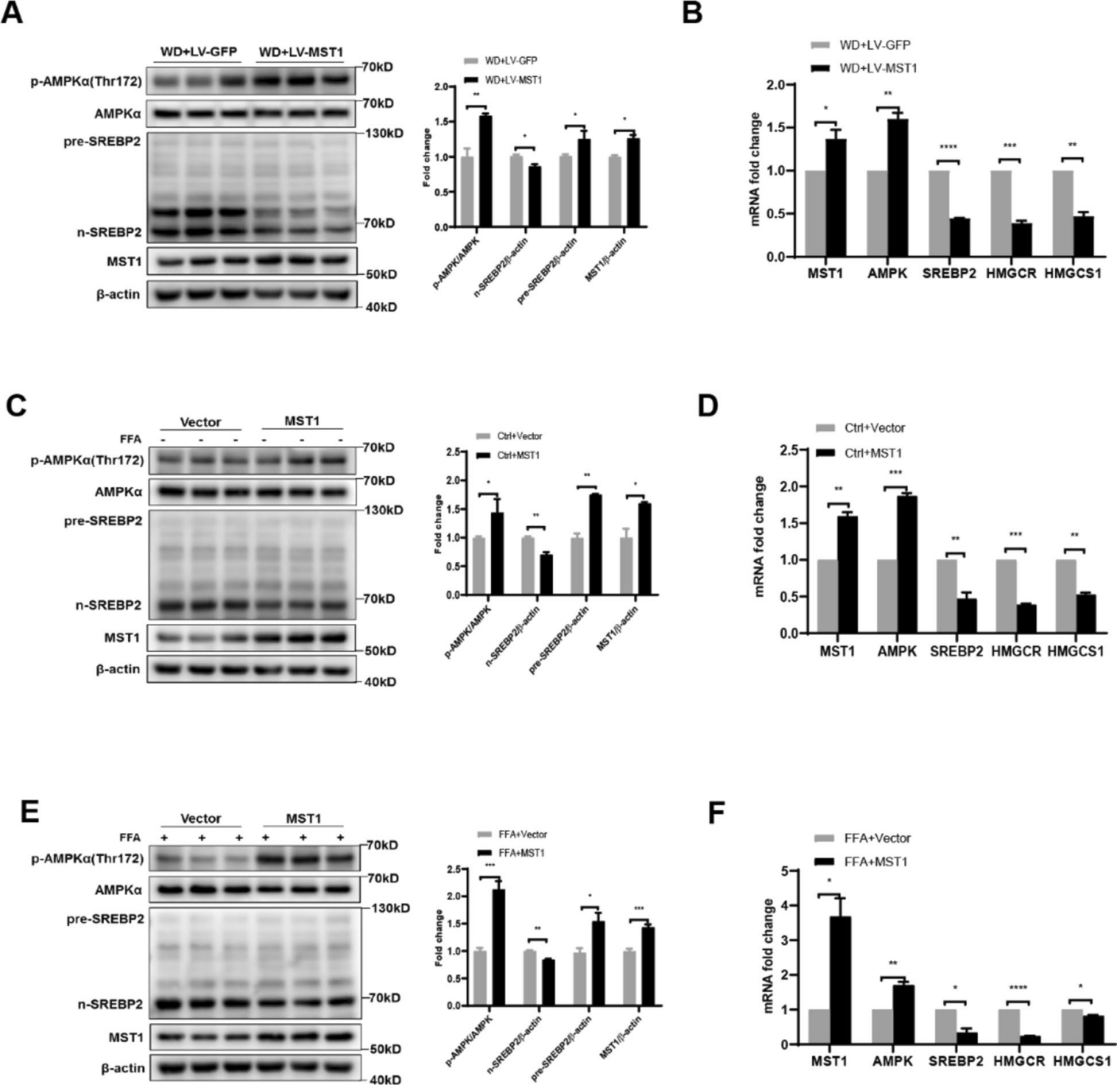
MST1 regulates cholesterol biosynthesis via modulation of the AMPK/SREBP2 axis. **a** Western blot analysis with densitometric quantification demonstrated hepatic expression profiles of *p*-AMPKα, AMPKα, nuclear SREBP2, precursor SREBP2, and MST1 in WD-fed C57BL6/J mice, including successful MST1 overexpression mediated by lentiviral delivery. **b** Quantitative analysis of mRNA levels revealed transcriptional patterns for MST1, AMPK, SREBP2, HMGCR, and HMGCS1 in hepatic tissue. **c** Immunoblotting results with corresponding densitometry displayed phosphorylation states of AMPKα and proteolytic processing of SREBP2 alongside MST1 overexpression in unstimulated HepG2 cultures. **d** Ectopic MST1 expression in baseline HepG2 cells significantly altered transcriptional activity of AMPK, SREBP2, and downstream cholesterol synthesis enzymes. **e** In FFA-challenged HepG2 models, immunoblot quantification illustrated MST1 overexpression effects on AMPK phosphorylation and SREBP2 maturation processes. **f** mRNA profiling in FFA-treated HepG2 cells confirmed regulatory impacts of MST1 overexpression on cholesterol metabolic markers. Data represent mean ± SEM from triplicate experiments* versus respective controls. *P < 0.05, **P < 0.01, ***P < 0.001, ****P < 0.0001

Given the established modulatory function of the AMPK/SREBP2 pathway in cholesterol homeostasis, this investigation demonstrated MST1 kinase-mediated phosphorylation of AMPK *α*. The study further examines MST1’s potential regulatory mechanism on cholesterol biosynthesis via this signaling axis. Immunoblot analysis of LV-MST1-treated C57BL6/J murine liver tissues revealed enhanced MST1 activity accompanied by elevated AMPK *α* Thr172 phosphorylation, subsequent AMPK activation, and suppressed SREBP2 maturation, ultimately reducing *n*-SREBP2 protein expression (Fig. 6a). Concurrent transcriptional profiling displayed upregulated AMPK mRNA levels contrasted with downregulated expression of SREBP2, HMGCR, and HMGCS1 genes (Fig. 6b). In vitro experiments using HepG2 cells with ectopically expressed MST1 plasmids under FFA-treated and basal conditions demonstrated MST1-mediated enhancement of AMPKα Thr172 phosphorylation, subsequent AMPK activation, and attenuation of SREBP2 proteolytic processing for nuclear translocation, consequently diminishing nuclear SREBP2 protein levels (Fig. 6c, e). Consistent with previous observations, transcriptional profiling revealed elevated AMPK mRNA abundance accompanied by reduced expression levels of SREBP2, HMGCR, and HMGCS1 transcripts following MST1 overexpression (Fig. 6d, f). By integrating in vitro and in vivo experimental data, it can be found that MST1 significantly activates the AMPK phosphorylation cascade reaction through the AMPK/mTORC1 signaling axis. It is worth noting that this regulatory mechanism can effectively block the protein hydrolysis process of SREBP2 and inhibit its nuclear translocation to the active form. Further analysis shows that MST1 not only reduces the expression level of SREBP2 transcription factor, but also specifically downregulates the mRNA abundance of key cholesterol metabolism genes such as HMGCR and LDLR. This series of regulatory effects ultimately leads to a significant inhibition of the overall activity of the cholesterol biosynthesis pathway.

## Discussion

NASH, an advanced phase of non-alcoholic fatty liver disease (NAFLD), has emerged as a growing public health challenge worldwide [60, 61]. Population-based studies estimate that nearly one-fourth of the adult population is affected by NAFLD, among whom over 20% develop NASH, significantly elevating the probability of hepatic cirrhosis and hepatocellular carcinoma progression [62, 63]. Research has shown that the core driving factor of NASH, liver cholesterol accumulation, can reach up to three times normal levels [11, 64]. By activating endoplasmic reticulum stress and NLRP3 inflammasome, it triggers hepatocyte pyroptosis and astrocyte activation, ultimately leading to irreversible fibrosis [65, 66]. Targeting the cholesterol metabolism regulatory network has become a key breakthrough. In NASH research, population-based cohort studies can be used for long-term patient follow-up. By collecting and analyzing data on factors such as cholesterol metabolism, inflammatory responses, and genetic background in a large group of NASH patients, we can gain a better understanding of the disease’s natural progression, identify high-risk populations, and develop more accurate predictive models for disease progression and prognosis [67]. MST1, as a key factor in metabolic regulation, inhibits adipogenesis by phosphorylating ACC1 and enhances mitochondrial fatty acid oxidation ability [34, 68]. It is worth noting that the MST1 deficiency model showed significant cholesterol accumulation, suggesting its involvement in cholesterol homeostasis regulation, but the specific mechanism is not yet clear [45]. This study constructs a liver specific gene modification model and combines multi omics analysis to systematically analyze the molecular pathways through which MST1 regulates cholesterol metabolism, providing theoretical support for the development of precision therapies for NASH based on new targets [69, 70].

Experimental analyses demonstrate that NASH models (both animal and cellular systems) exhibit concurrent activation of cholesterol biosynthesis pathways, hepatic FC accumulation, and progressive inflammatory/fibrotic responses, accompanied by diminished MST1 expression. Mechanistic studies reveal MST1 deficiency promotes SREBP2 nuclear localization, upregulates cholesterologenic enzymes, exacerbates hepatocellular cholesterol overload, and intensifies NASH-associated hepatic injury and fibrogenesis. The regulatory mechanism involves MST1-mediated phosphorylation at the AMPKα Thr172 residue. Ectopic MST1 expression enhances AMPKα Thr172 phosphorylation, impedes SREBP2 nuclear translocation, suppresses cholesterol biosynthesis transcriptional programs, reduces hepatic cholesterol deposition, and mitigates NASH-related inflammatory responses and extracellular matrix remodeling.

Lipidomics analysis of human liver tissue showed that compared to NAFL individuals, FC levels were significantly elevated in NASH patients [71]. In NAFLD patients, NASH occurs simultaneously with fibrosis, parallel to the accumulation of FC in the liver. Artificially induced hepatic FC accumulation exacerbates inflammatory infiltration and fibrogenesis, whereas therapeutic reduction of pathological FC burden demonstrates NASH attenuation potential [72]. Pathological analyses of clinical NASH specimens and HFHC-diet murine models reveal cholesterol crystallization within hepatocyte lipid droplets triggers KC activation, instigating pro-inflammatory cytokine cascades (TNF-*α*, IL- 1β, IL- 6) that drive CCL2/CCR2-mediated neutrophil recruitment and monocyte trafficking [18]. Differentiated pro-inflammatory macrophages sustain hepatic inflammation, while TGF-*β*-mediated activation of hepatic stellate cells triggers myofibroblast differentiation and extracellular matrix deposition. [73–75]. The present investigation documented concordant pathological manifestations in HFHC-induced murine NASH models, quantitatively reinforcing the mechanistic linkage between FC overload and inflammatory liver injury progression. Emerging technologies such as spatial transcriptomics, combined with spatial data analysis methods in bioinformatics, are revolutionizing oncology research. These technologies enable a comprehensive analysis of gene expression distribution within the tumor microenvironment, accelerating the advancement of translational oncology [76]. Mechanistic studies have shown that components of the Hippo signaling pathway, particularly the LATS1/2 kinases and the YAP and TAZ transcriptional regulators, play a critical role in regulating liver cholesterol homeostasis. Research has shown that the potential to respond to various biological reactions has been demonstrated through the regulation of specific biomolecules and the application of specific compounds [77]. This study identifies MST1 as a critical regulator of hepatic cholesterol homeostasis, revealing its therapeutic potential for NASH through mechanistic insights. Given the liver’s central role in sterol metabolism, NAFLD progression involves multiple patterns of cholesterol dysregulation that lead to hepatic cholesterol sequestration. Pathophysiological analyses link hyperinsulinemia and chronic inflammation to impaired SCAP/SREBP2 regulatory control, driving pathological cholesterol accumulation in murine models [78–80]. Clinical investigations demonstrate that NAFLD/NASH patients exhibit enhanced nuclear SREBP2 localization, accompanied by increased HMGCR transcriptional/translational activity and phosphorylation, while showing reduced LDLR expression and impaired ABC transporter (ABCA1, ABCG1, ABCG5) function. Experimental data from NASH murine models revealed significant elevations in serum TC, LDL, and hepatic TC/FC concentrations. MST1-deficient murine models exhibit profound lipid dysmetabolism attributed to reduced hepatic Sirt1 expression, amplified ubiquitin–proteasome degradation mechanisms, and elevated SREBP- 1c activity [35]. Contemporary systems biology approaches prove instrumental in decoding multifactorial disease pathogenesis and innovating therapeutic interventions [81–83]. Mechanistic studies delineate Hippo signaling components—particularly LATS1/2 kinases, YAP, and TAZ transcriptional regulators—as critical modulators of hepatic cholesterol dynamics. Experimental evidence demonstrates cholesterol overload-induced calcium signaling activation stimulates RhoA-mediated LATS1/2 inhibition, culminating in transcriptional upregulation of TAZ/YAP proteins that drive NASH-associated fibrogenesis [84]. YAP further functions as a transcriptional coactivator for SREBP- 1c and SREBP- 2, directly governing FAS and HMGCR expression. Therapeutic LATS1 overexpression impedes YAP dephosphorylation and nuclear translocation, ameliorating hepatic steatosis and systemic insulin resistance in diabetic models [85]. Biochemical investigations demonstrate LATS2 binds to endoplasmic reticulum-associated SREBP- 1/2 precursors, inhibiting proteolytic processing and attenuating SREBP nuclear transcriptional activity [86]. Emerging evidence suggests MST1 may functionally intersect with PPARγ and LXR signaling networks—pivotal regulators of hepatic lipid homeostasis. Exploring these interactions offers vital insights into MST1’s multipronged modulation of metabolic pathways [42, 43]. Collective findings establish Hippo pathway effector MST1 as a nexus integrating hepatic lipid/cholesterol metabolism with NASH pathogenesis. Building upon prior mechanistic work, this study delineates MST1-mediated AMPK Thr172 phosphorylation as a regulatory checkpoint for sterol biosynthesis. Pharmacological AMPK activation via AICAR in MST1-deficient models rescues hepatic FC overload and injury, confirming AMPK as a downstream MST1 substrate. Mechanistically, AMPK governs SREBP2 nuclear translocation efficiency and transcriptionally modulates SREBP2-HMGCR/HMGCS1 axis activity, aligning with established metabolic regulatory paradigms [59, 87, 88]. Experimental induction of MST1 overexpression across complementary in vivo and in vitro systems systematically mapped the AMPK/SREBP2 signaling cascade dynamics triggered by MST1-dependent AMPKα Thr172 phosphorylation. This kinase activity modulates SREBP2 nuclear trafficking efficiency, subsequently altering the transcriptional output of SREBP2 and its cholesterologenic targets HMGCR and HMGCS1. The resultant suppression of sterol biosynthesis alleviates hepatocellular FC burden, consequently ameliorating NASH-associated inflammatory infiltration and extracellular matrix remodeling severity.

However, current research still has certain limitations, as the pathological heterogeneity between animal models and human diseases limits translational studies. The proportion of immune cells and fibrosis processes in the model system differ significantly from clinical samples; Meanwhile, the spatiotemporal dynamic characteristics of AMPK activation have not been fully analyzed, and the differences in regulatory patterns between early and late stages of the disease may affect the accuracy of target intervention strategies [89, 90]. These limitations suggest that extrapolation of existing conclusions should be cautious and further validated through models that are closer to human pathological features [91, 92]. The integration of modern technologies with traditional Chinese medicine offers novel perspectives and possibilities for drug development, particularly in addressing the multifaceted pathology of NASH [93, 94]. In this context, organoid technology is transitioning from laboratory research to clinical applications. By constructing disease-specific models integrated with bioinformatics analysis platforms, this approach enables systematic dissection of pathogenic mechanisms and drug response profiles, offering new strategies to overcome the heterogeneity bottlenecks of traditional models [95]. Building on this, future efforts could engineer humanized organoid models incorporating diverse cell types to recapitulate the complex microenvironment of NAFLD, thereby validating MST1’s regulatory role and bridging foundational research with clinical translation [96]. Design allosteric modulators based on the structural features of MST1, and validate their stage specific therapeutic effects through a multidimensional efficacy evaluation system [97, 98]. However, the clinical translation of these strategies still requires overcoming technical challenges such as data privacy protection, cross-center model generalizability, and real-time analysis. Future efforts could explore the integration of federated learning frameworks and edge computing technologies to optimize the coordinated implementation of dynamic monitoring and personalized interventions [99, 100]. Collectively, these methodological advances not only deepen our understanding of cellular differentiation, signal transduction, and stromal interactions, but also establish a pivotal framework for developing novel therapeutic strategies, thereby propelling the evolution of precision medicine and regenerative medicine [101–103]. In brief, this study reveals that MST1 markedly reduces the accumulation of FC in the liver by activating the AMPK/SREBP2 signaling axis, thereby effectively impeding the pathological progression of NASH. These findings collectively underscore MST1 as a promising therapeutic target for modulating NAFLD/NASH progression through its regulatory effects on cholesterol biosynthetic pathways and inflammatory–fibrotic cascades. Future research will delve into the interaction between MST1 and other metabolic pathways, comprehend its role in human models, and assess MST1-targeted interventions in clinical scenarios, facilitating the transition of MST1 from fundamental mechanisms to clinical treatment and offering a novel paradigm for precise intervention of NAFLD [104, 105].

## Conclusion

In conclusion, our study demonstrates that MST1 can alleviate hepatic free cholesterol load and thus mitigate the progression of NASH through the regulation of AMPK/SREBP2 signaling. This highlights MST1 as a promising target for the treatment of NAFLD/NASH. Future research will be crucial in exploring MST1’s interactions with other metabolic pathways, understanding its role in human models, and evaluating the long-term therapeutic potential of MST1-targeted interventions in clinical settings.

## Supplementary Information

Additional file 1

## Data Availability

No datasets were generated or analysed during the current study.

## Abbreviations

AICAR: AMPK activator
ALT: Alanine aminotransferase
AMPK: 5′ AMP-activated protein kinase
AST: Aspartate aminotransferase
CCL2: Chemokine C–C motif ligand 2
FC: Free cholesterol
FFA: Free fatty acids
H&E: Haematoxylin and eosin
HDL-C: High-density lipoprotein cholesterol
HMGCR: 3-Hydroxy- 3-methyl-glutaryl-coenzyme A reductase
HMGCS1: 3-Hydroxy- 3-methylglutaryl coenzyme A synthetase1
HSCs: Hepatic stellate cells
IL- 1β: Interleukin- 1β
IL- 6: Interleukin- 6
KCs: Kupffer cells
LDs: Lipid droplets
LDL-C: Low density lipoprotein cholesterol
MST1: Mammalian sterile 20-like kinase 1
NASH: Non-alcoholic steatohepatitis
NCD: Normal chow diet
SREBP2: Sterol regulatory element-binding transcription factor 2
TC: Total cholesterol
TGF-*β*: Transforming growth factor-beta
TNF-*α*: Tumor necrosis factor-alpha
WD: Western diet
WT: Wild-type
