# Supplementary material for: Mammalian Ste20-like kinase 1 regulates AMPK to mitigate the progression of non-alcoholic fatty liver disease
Source: Eur J Med Res. 2025 Apr 17;30:296. doi: 10.1186/s40001-025-02557-9 (PMC12004885; doi:10.1186/s40001-025-02557-9)
Supplement: Supplementary file 1 — Additional file 1 [file 40001_2025_2557_MOESM1_ESM.docx]

**Supplementary figures**

**
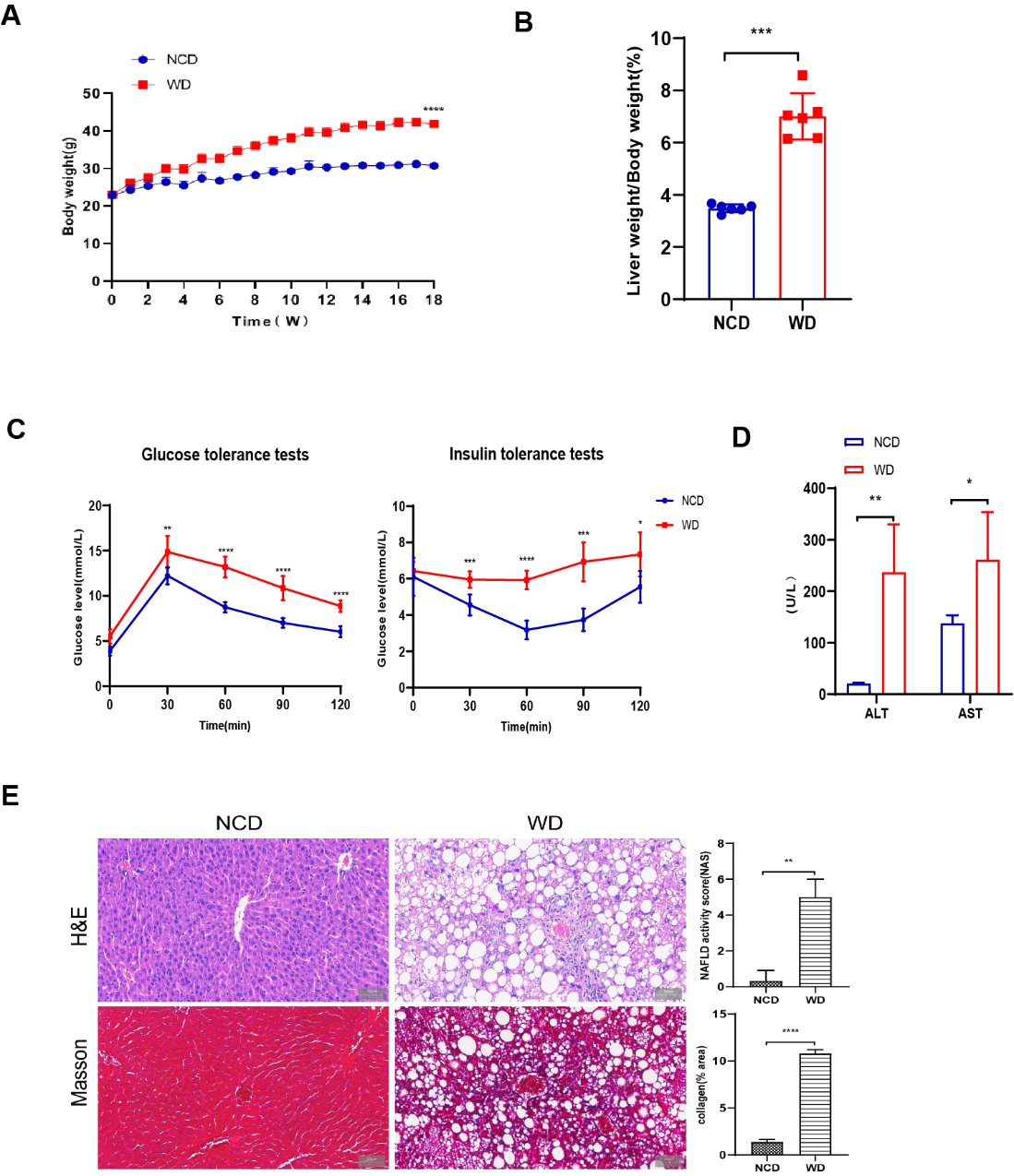
Supplementary figure 1.** Establishment of the NASH mouse model. WTC57BL6/J mice were subjected to NCD and WD diets for 18 weeks. (A) Mouse body weight. (B) Liver wet weight of mice. (C) Glucose tolerance and insulin tolerance tests in mice. (D) Serum ALT and AST levels in mice. (E) Representative H&E and Masson's trichrome staining of liver sections (200×). Data are presented as the mean ± SEM. n = 6-8 mice/group. *P < 0.05, **P < 0.01, ***P < 0.001, ****P < 0.0001 vs the designated two groups.


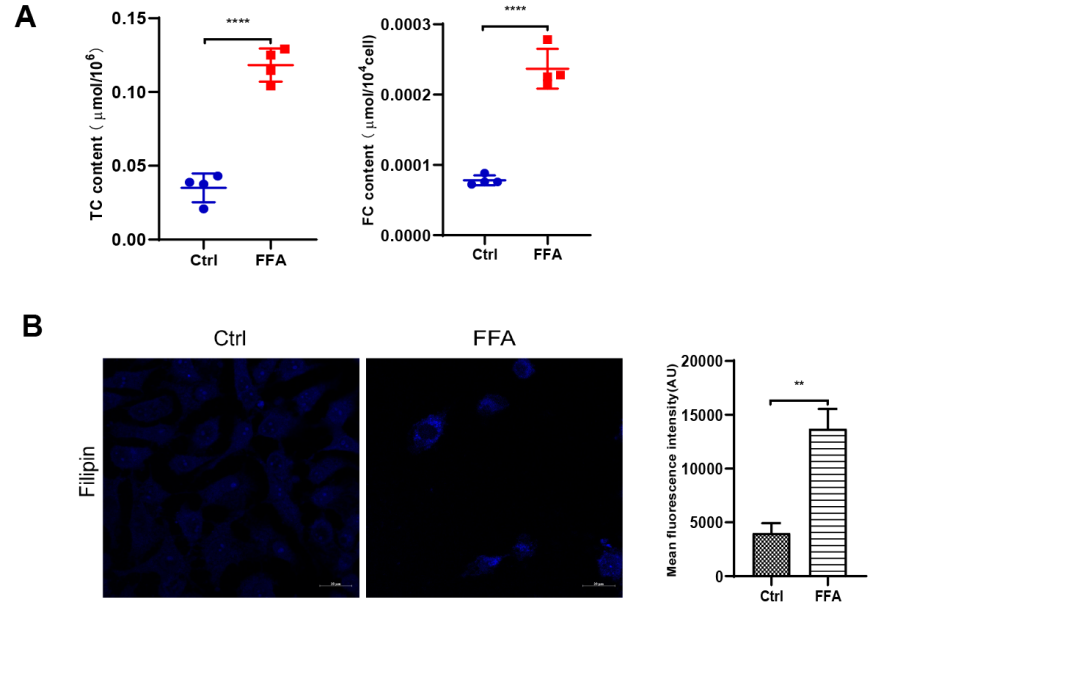


**Supplementary figure 2.** Increased cholesterol accumulation in vitro NASH cell model. HepG2 cells were induced with 1mM FFA for 24 hours to establish the in vitro cellular model. (A) Measurement of cellular TC and FC content. (B) Representative cellular Filipin staining illustrating cellular membrane FC accumulation (scale bars, 20 μm). Data are presented as the mean ± SEM. n = 3 independent experiments. *P < 0.05, **P < 0.01, ***P < 0.001, ****P < 0.0001 vs the designated two groups.

**
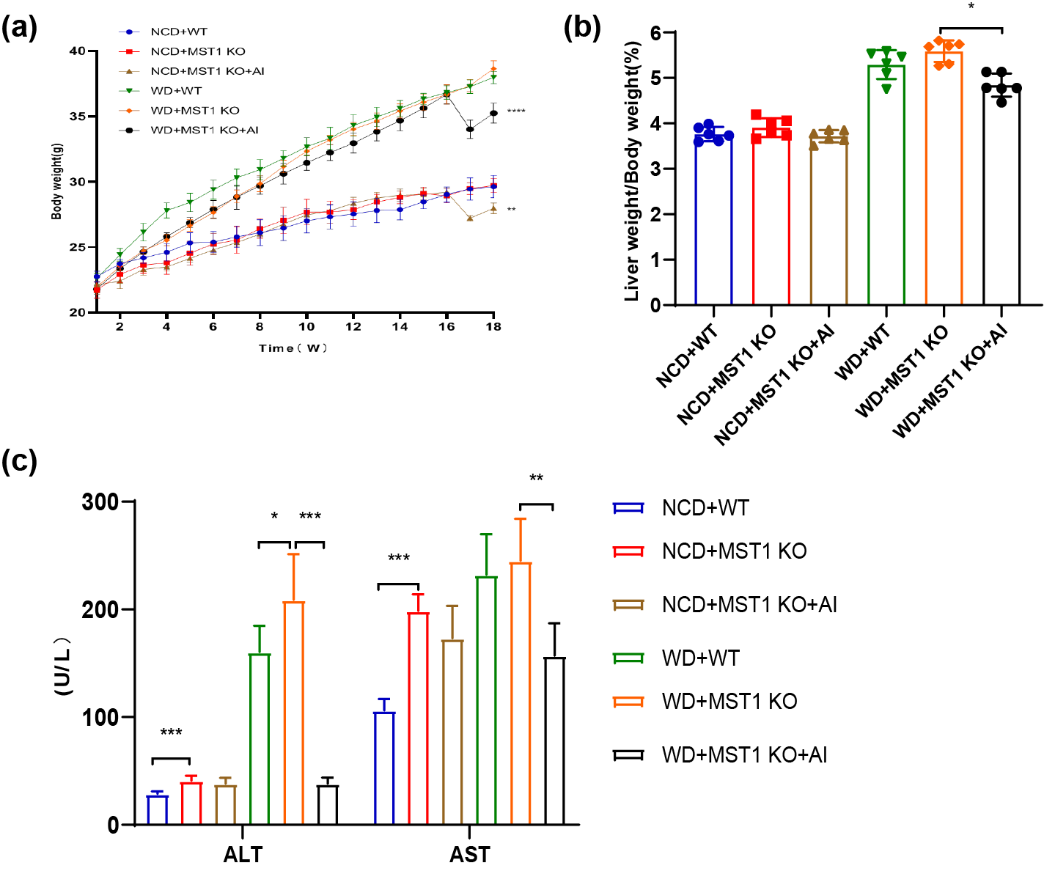
**

**Supplementary figure 3.** MST1 deficiency results in hepatic cholesterol accumulation and liver injury; AMPK activation improves liver injury. MST1-KO mice were fed either NCD or WD diets for 16 weeks, after which one group of MST1-KO mice received AICAR treatment for 2 weeks. (A) Mouse body weight. (B) Liver wet weight. (C) Serum ALT and AST levels. Data are presented as the mean ± SEM. n = 6 mice/group. **P < 0.05, **P < 0.01, ***P < 0.001, ****P < 0.0001 vs the designated two groups.


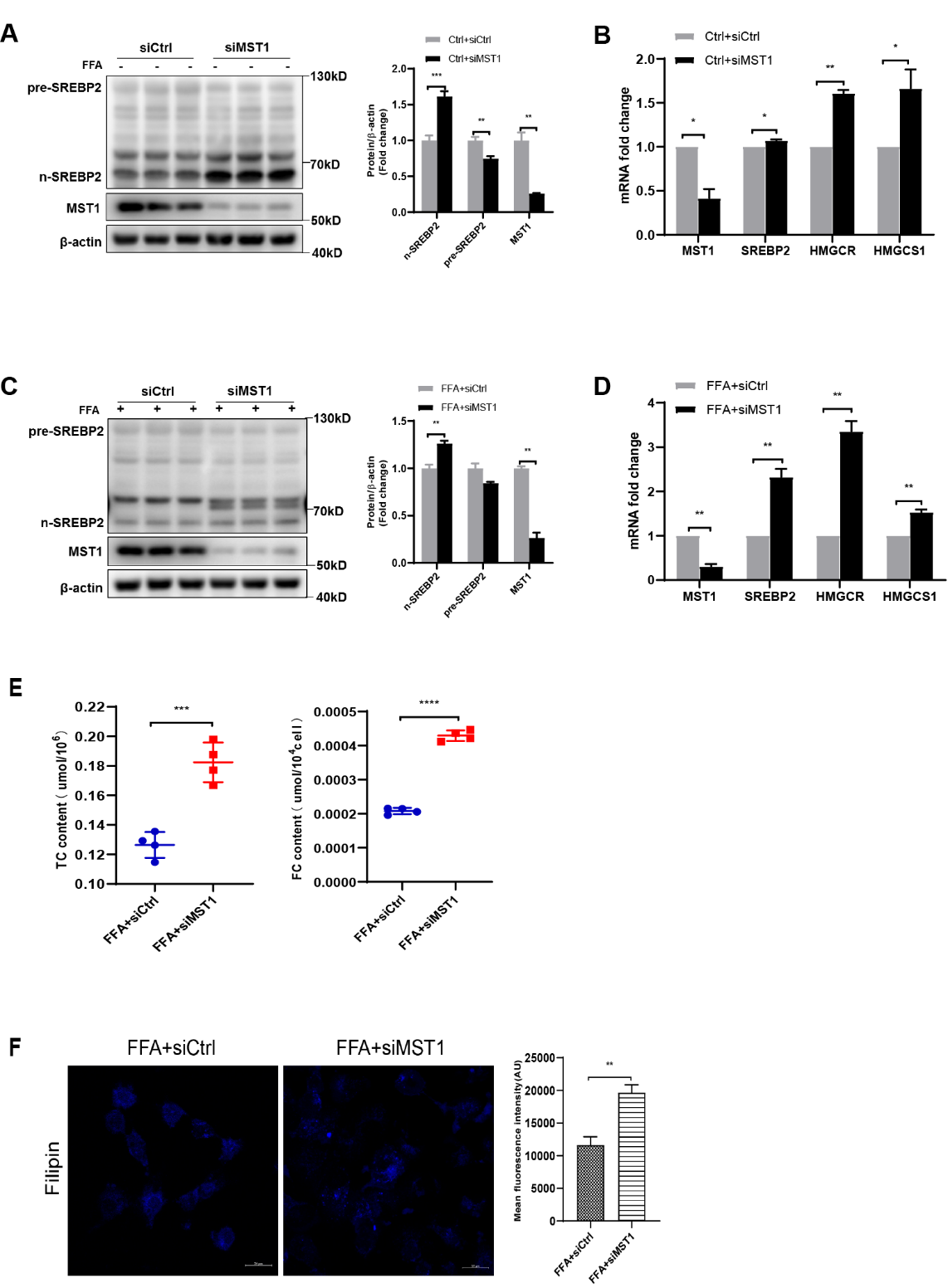


**Supplementary figure 4.** Reduced MST1 activity in the cholesterol synthesis pathway exacerbates cholesterol accumulation in NASH hepatic cells. HepG2 cells induced or not induced by FFA were subjected to MST1 silencing using siRNA. (A) Immunoblotting and grayscale analysis of n-SREBP2, pre-SREBP2, and MST1 protein expression in non-induced HepG2 cells. (B) mRNA expression levels of MST1, SREBP2, HMGCR, and HMGCS1 in non-induced HepG2 cells. (C) Immunoblotting and grayscale analysis of n-SREBP2, pre-SREBP2, and MST1 protein expression in FFA-induced HepG2 cells. (D) mRNA expression levels of MST1, SREBP2, HMGCR, and HMGCS1 in FFA-induced HepG2 cells. (E) Measurement of TC and FC content in the NASH cell model. (F) Representative Filipin staining displaying FC deposition in the NASH cell model (scale bars, 20 μm). Data are presented as the mean ± SEM. Data represent 3 independent experiments. *P < 0.05, **P < 0.01, ***P < 0.001, ****P < 0.0001 vs the designated two groups.

**
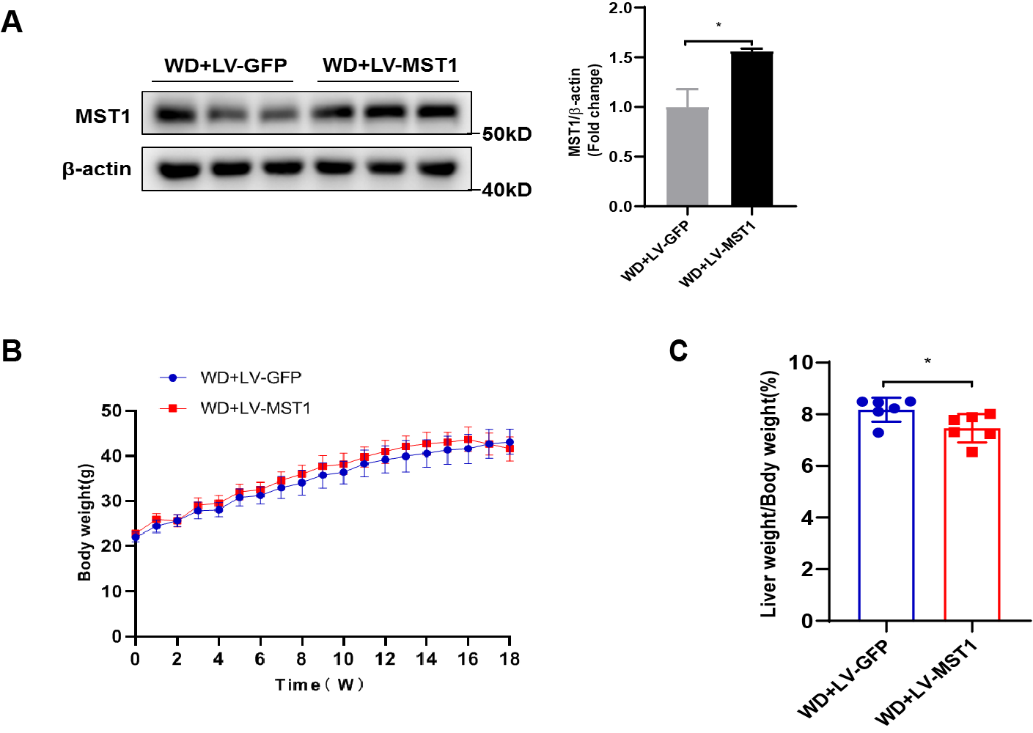
**

**Supplementary figure 5.** MST1 is capable of ameliorating hepatic cholesterol deposition. C57BL/6J mice were fed a WD for 16 weeks, followed by treatment with an MST1-overexpressing lentivirus. (A) Western blot and densitometry analysis revealed the expression levels of MST1 in the livers of WD-fed mice. (B) Changes in body weight of the mice. (C) Wet liver weight of the mice. Data are presented as the mean ± SEM. n = 6 mice/group. *P < 0.05 vs the designated two groups.
